# Functional improvement in chronic human spinal cord injury: Four years after acidic fibroblast growth factor

**DOI:** 10.1038/s41598-018-31083-4

**Published:** 2018-08-23

**Authors:** Chin-Chu Ko, Tsung-Hsi Tu, Jau-Ching Wu, Wen-Cheng Huang, Yun-An Tsai, Shih-Fong Huang, Hsueh-Chen Huang, Henrich Cheng

**Affiliations:** 1 Jhong Jheng Spine & Orthopedic Hospital, Kaohsiung, Taiwan; 2 Department of Neurosurgery, Neurological Institute, Taipei Veterans General Hospital, Taipei, Taiwan; 3 Center for Neural Regeneration, Neurological Institute, Taipei Veterans General Hospital, Taipei, Taiwan; 4 Department of Physical Medicine and Rehabilitation, Taipei Veterans General Hospital, Taipei, Taiwan; 5 School of Medicine, National Yang-Ming University, Taipei, Taiwan; 6 Institute of Pharmacology, National Yang-Ming University, Taipei, Taiwan; 7 Taiwan International Graduate Program in Molecular Medicine, National Yang-Ming University and Academia Sinica, Taipei, Taiwan

## Abstract

Few treatments have proven effective for patients with chronic spinal cord injury (SCI). This study aimed to evaluate the efficacy and safety of acidic fibroblast growth factor (aFGF) in human SCI. This was an open-label prospective clinical trial of aFGF with an extended follow-up to 48 months. All patients were treated with aFGF 3 times, including once directly applied to the injured spinal cord during neurolysis surgery, and twice via lumbar punctures at 3- and 6-months post-operation. Every patient was evaluated with standardized measurements of neurological functions. The trial initially enrolled 60 patients (30 cervical and 30 thoracolumbar SCI), but only 46 (21 cervical- and 25 thoracolumbar-SCI) completed the follow-up. The ASIA impairment scales, motor, pin prick, light touch, and FIM motor subtotal scores were all improved in both groups, except that the ASIA scores of light touch only demonstrated tendency of increase in the cervical-SCI group. All patients had a decrease in dependence, and there were no major adverse events or other oncological problems throughout the follow-up. At 48 months, the study demonstrated that aFGF was safe, feasible, and could yield modest functional improvement in chronic SCI patients. Further randomized control investigations are warranted for validation of its optimal dosage.

## Introduction

Spinal cord injury (SCI) could be a devastating event and might tremendously affect the patients and their family, both physically and psychologically. It may result in a loss of sensory, motor or autonomic nerve functions, leading to life impairment and loss of quality of life. Early surgical decompression and rehabilitation remain the only proven guidelines until now^1,2^. There has been little advance in the aspect of clinical pharmacology^3^.

Cheng *et al*. enlightened researchers on the issue of central neural regeneration with the treatment of complete transection of the spinal cord of rats^4^. The strategy of peripheral nerve grafting together with acidic fibroblast growth factor (aFGF) in fibrin glue was further proven to benefit the rodents with spinal cord injuries and cervical root injuries^5–12^. Since aFGF was found to be a normal spinal cord constituent and was released only after damage, it was believed to be involved in nerve repair^4,13,14^. A recent study demonstrated the neuroprotection and axonal regeneration and remyelination from the overexpression of aFGF in spinal cords following SCI, suggesting the positive role of aFGF in functional recovery^15^. The clinical application of aFGF, with or without nerve grafting, was also reported to advantage neurological function improvement after spinal cord injuries, cervical root injuries, brachial plexus injuries, and common peroneal nerve injuries in human patients^16–22^. Based on these inspiring results, a prospective clinical trial about the application of aFGF to injured spinal cords was conducted^23^. Unlike the animal models, patients in the clinical scenario rarely sustained discontinuity of neural structure; therefore, nerve grafting was not used in the majority of patients in that trial. The mid-term (24-month) results reported the positive conclusions that the use of aFGF for spinal cord injury was safe and feasible, and that there were significant improvements in neurological evaluation scores and functional independence measures at 24 months after treatment^23^.

After that, the objectives were consecutively observed for 2 more years with no further intervention. As an extension of the trial, the current study aims to evaluate the long-term efficacy and safety of aFGF usage in combination with surgical intervention for human spinal cord injury.

## Methods

### Study Design

A prospective, open-label, uncontrolled clinical trial concerning human spinal cord injury was conducted. The mid-term results at 24 months have been previously reported^23^. The current study was an extension of the trial (ClinicalTrials.gov Identifier: NCT03229031, 25/07/2017). The materials and methods of the trial were detailed in the earlier publication, and are briefly summarized as below^23^.

### Inclusion, Exclusion, and Withdrawal

The enrolled cases were patients suffering from spinal cord injury for more than 10 weeks in duration, with a relatively stable neurological status. The neurological status was determined according to at least 2 separate assessments with an interval of 6 weeks. The age of the patients ranged from 16 to 68 years. Informed consent from each of the participants was obtained.

Patients with major medical diseases or malignancy histories were excluded. Those who were ventilator-dependent due to spinal cord injury, or who had a chest/lung injury or brain injury were excluded. To control the heterogeneity, patients with myelomalacia greater than 2 cm on MRI were excluded. Those with inadequate psychological status or an inability to cooperate with multiple assessments were also excluded.

Patients withdrew from the trial due to major adverse events, noncompliance, failure to accomplish follow-up assessment, or one’s will to discontinue. The trial was approved by the Department of Health of Taiwan. All methods were performed in accordance with the relevant guidelines and regulations.

### Surgical Procedure

The surgical purpose was to apply the aFGF directly to the injured neural tissues and neurolysis. The patients underwent total laminectomy at the injured spinal segments. The dura was incised at midline to expose the spinal cord and rootlets. Meticulous neurolysis was done. The dentate ligaments were transected for advanced neurolysis and extensive decompression from scar tissue.

Continuity of the neural structure was particularly inspected. No peripheral nerve grafting was done unless apparent discontinuity of the spinal cord was noted. A therapeutic cocktail was applied to the injured neural tissue after neurolysis. The therapeutic cocktail was a 2-ml combination of aFGF (20 µg/dose, corresponding to 400U/dose) and fibrin glue (Beriplast^®^ P)^24^, with the same concentration regimen described in previous reports^4,21–23^. The aFGF was bound in the fibrin glue and kept localized *in situ*, and was gradually released^4,25^. Duraplasty was performed and fibrin glue was applied to prevent postoperative cerebral spinal fluid leakage.

### Therapeutic Agent

The first dose of aFGF in fibrin glue was given during the surgery, as mentioned above. All patients received the other 2 adjuvant boosters of 2 ml combination of aFGF and fibrin glue via lumbar puncture at 3 and 6 months postoperatively. The human aFGF, produced in *Escherichia coli* (that is, BL21[DE3]), was a single, nonglycosylated, polypeptide chain with 135 amino acids and molecular mass of 15281 D. It was purified by 3 columns: ion exchange, affinity (that is, heparin column), and gel extraction. Purity was greater than 99% by RP-HPLC (reverse-phase high-performance liquid chromatography) analysis and SDS-PAGE (sodium dodecyl sulfate polyacrylamide gel electrophoresis). The dosage applied in this trial was the same as in previously published clinical trials^21–23^.

### Rehabilitation Program

All patients underwent a comprehensive rehabilitation program after the surgery as soon as tolerable and kept it up for 2 years. The program consisted of bowel movement training, urinary training, sensory and motor function, and physical and occupational therapy.

### Outcome Measurement

Two independent staff members (physical therapists or physical medicine and rehabilitation physicians) performed the evaluation preoperatively at 3, 6, 12, 18, 24 and 48 months postoperatively. The measurement items included the American Spinal Injury Association (ASIA) Impairment Scale, motor score, sensory score, and overall neurological level, and Functional Independence Measure (FIM)^26,27^. The data were recorded by a third party. The details are described in the previous report^23^.

### Adverse Events

All events of newly developed or aggravated previously existing diseases were monitored and reported. They were classified and coded under the system of the Medical Dictionary of Regulatory Activities (MedDRA). The adverse events were classified into mild, moderate, and severe. The relationship of adverse events to the patient’s condition was classified into 5 levels of “not,” “unlikely,” “possible,” “probable,” and “definitely” related.

### Data Processing and Statistics

All data were collected, monitored simultaneously, analyzed by a contract research organization (Statplus, Inc.), and verified. Statistical software (version 9.1.3, SAS Institute, Inc.) was used. Missing data from early withdrawals or dropouts were processed using the method of last value carried forward (LOCF). The Wilcoxon signed rank test was used to compare numerical data, while the McNemar’s test was used for categorical data.

## Results

### Patient Demographics

**Figure 1:**
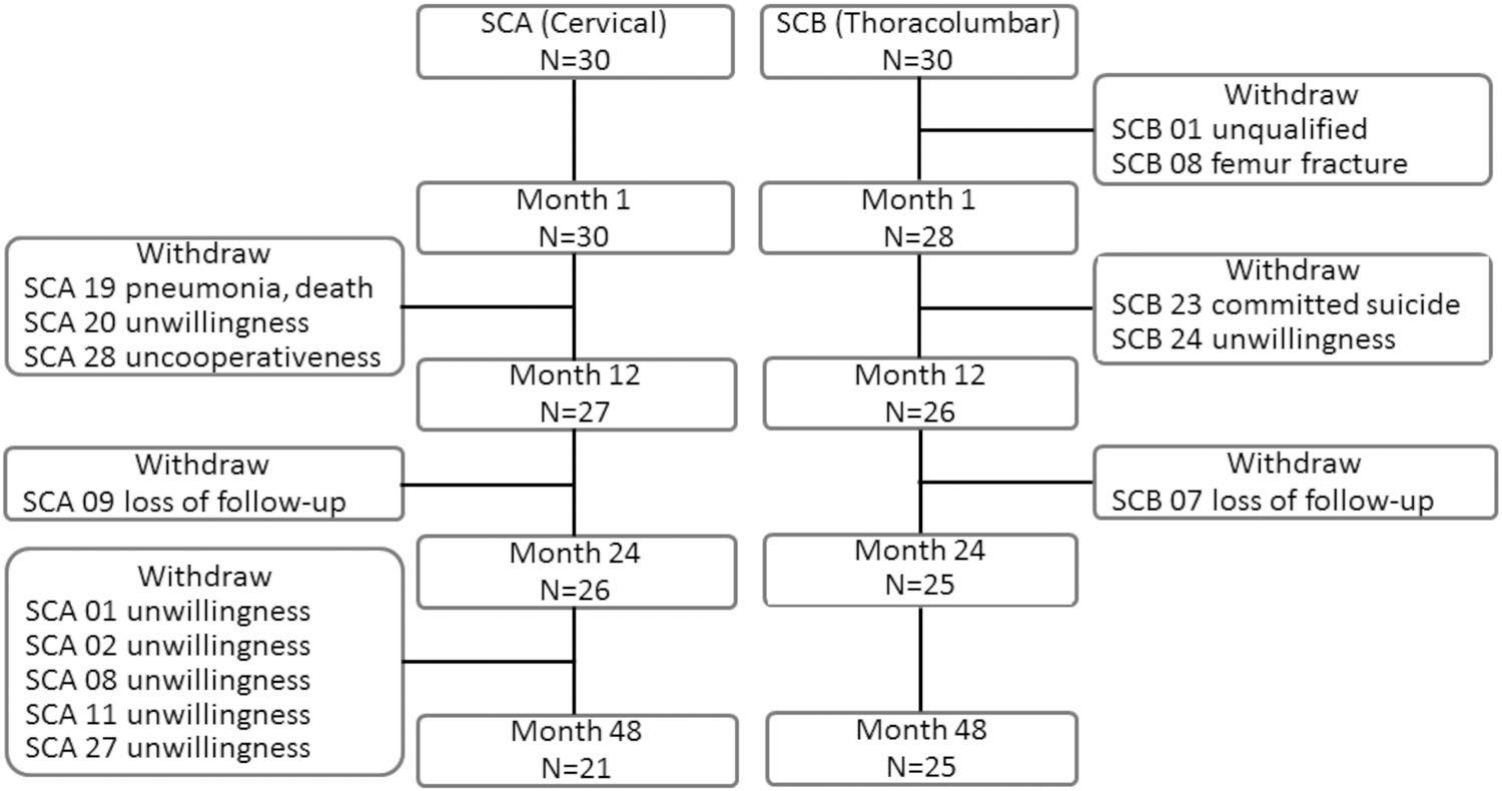
Algorithm of enrollment and withdrawal. N = number of patients; SCA = cervical SCI group; SCB = thoracolumbar SCI group.

The clinical trial enrolled a total number of 60 participants (45 male and 15 female), divided into cervical (30 patients) and thoracolumbar (30 patients) groups based on the injury level. The characteristics of the initially enrolled 60 patients and the distribution of their SCI levels were previously reported^23^. At the time point of 48-months follow-up, 46 patients (21 in the cervical group and 25 in the thoracolumbar group) remained in the trial. Withdrawals were due to refusal, unrelated adverse events, or failure to participate in the planned rehabilitation (Fig. 1).

The remaining 46 patients consisted of 35 males and 11 females, with a mean age of 35.7 ± 15.1 years (mean ± SD). The causes of injury included motor vehicle accidents (43.5%), fall from a height (26.1%), sports (6.5%), and others (23.9%). The vast majority of the patients (95.7%) had undergone decompression and/or fixation surgery prior to partaking in the trial. At presentation, the ASIA Impairment Scale was mainly graded A (67.4%), followed by B (15.2%), C (15.2%), and D (2.2%). Between the initial injury and the trial, there was a duration of 26.2 ± 26.9 months (mean ± SD), with a range of 3 and 136 months (Table 1).

**Table 1:** Characteristics of 46 patients with SCI followed for 48 months.

Variable	Value^**†**^		
	Cervical SCI	Thoracolumbar SCI	Overall
Number of patients	21	25	46
Sex			
male	17 (81.0)	18 (72.0)	35 (76.1)
female	4 (19.0)	7 (28.0)	11 (23.9)
Age (years)	38.8 ± 16.9	33.1 ± 13.1	35.7 ± 15.1
mean ± SD (range)	(17–65)	17–68)	(17–68)
Follow-up (months) (mean ± SD)	51.8 ± 2.9	48.3 ± 4.6	49.9 ± 4.3
Cause of SCI			
motor vehicle accident	10 (47.6)	10 (40.0)	20 (43.5)
fall	4 (19.1)	8 (32.0)	12 (26.1)
sports	3 (14.3)	0 (0.0)	3 (6.5)
other	4 (19.1)	7 (28.0)	11 (23.9)
Preceding decompression/fixation operation			
no	2 (9.5)	0 (0.0)	2 (4.4)
yes	19 (90.5)	25(100.0)	44 (95.7)
ASIA Impairment Scale			
A	8 (38.1)	23 (92.0)	31 (67.4)
B	7 (33.3)	0 (0.0)	7 (15.2)
C	5 (23.8)	2 (8.0)	7 (15.2)
D	1 (4.8)	0 (0.0)	1 (2.2)
Duration (months)^**‡**^	27.4 ± 26.0	25.2 ± 28.1	26.2 ± 26.9
mean ± SD (range)	(3–105)	(3–136)	(3–136)

^**†**^Unless otherwise indicated, values represent the number of patients with percentages in parentheses.

^**‡**^Interval between the injury and the repair operation.

### ASIA Impairment Scale

In the cervical group, 4 of the 21 (19%) patients obtained improvement on the ASIA Impairment Scale. Among them, 3 of the 8 patients who initially presented as Grade A improved to Grade B (1 patient) and Grade C (2 patients), respectively, at 48-months follow-up. Besides, of the 5 patients initially Graded C, 1 patient improved to Grade D, while 1 patient deteriorated to Grade B. The other 1 of the 7 patients with an initial Grade B deteriorated to Grade A. The overall ASIA Impairment Scale changed significantly at 48 months (*p* = 0.001) (Table 2).

**Table 2:** Comparison of ASIA Impairment Scale between baseline and 48 months postoperatively in each group^†^.

ASIA Impairment Scale		48 months after aFGF						P value^‡^
		A	B	C	D	E	Total	
Baseline for Cervical SCI	A	5	**1**	**2**			8	0.001*
	B	**1**	6				7	
	C		**1**	3	**1**		5	
	D				1		1	
	E						0	
	Total	6	8	5	2	0	21	
Baseline for Thoracolumbar SCI	A	11	**12**				23	0.002*
	B						0	
	C			1	**1**		2	
	D						0	
	E						0	
	Total	11	12	1	1	0	25	

^**†**^Values in bold face represent the numbers of patients whose ASIA Impairment Scales improved from the baseline.

^**‡**^Based on Pearson Chi-Square Test.

*Significant at p < 0.05.

Regarding the thoracolumbar group, 13 of the 25 (52%) patients achieved improvement. Among them, 12 of the 23 patients with an initial Grade A improved to Grade B at 48-months follow-up. One of the 2 patients with Grade C improved to Grade D. There was no deteriorating case in this subgroup. The overall ASIA Impairment Scale improved significantly at 48 months (*p* = 0.002) (Table 2).

### ASIA scores and neurological levels

**Figure 2:**
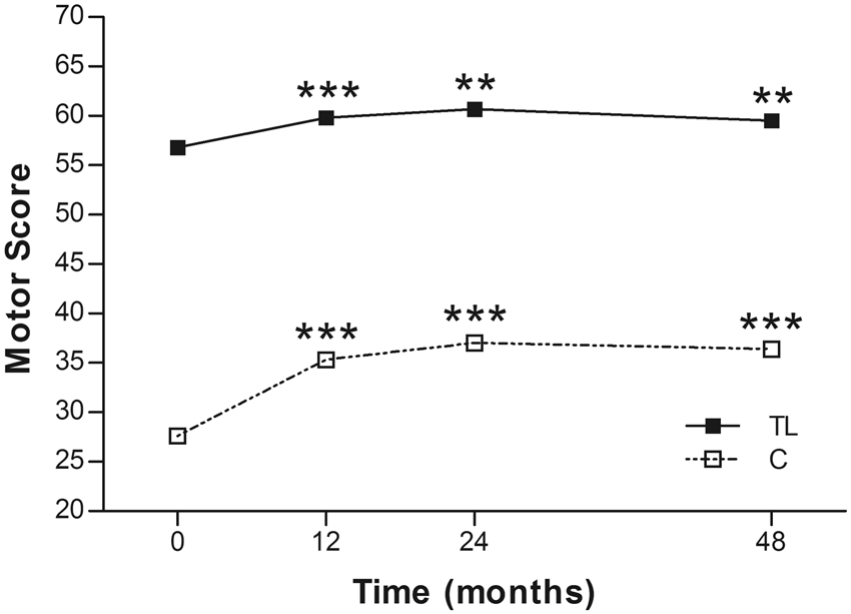
Mean ASIA motor scores of the 2 groups at each time point. C = cervical group; TL = thoracolumbar group; **p < 0.005; ***p < 0.0001.

The ASIA scores improved significantly in both groups at 48 months post operation. The motor scores improved from 27.6 ± 15.6 to 36.4 ± 17.6 in the cervical group (p < 0.0001), and from 56.8 ± 9.2 to 59.5 ± 8.5 in the thoracolumbar group (p < 0.0001) (Table 3) (Fig. 2). The improvement was similar to that at 24 months post operation. There was no further improvement from month 24 to 48 post operation. However, it should be noted that there were no additional aFGF treatment administration, either.

**Table 3:** Motor score, sensory score, and neurological level in each group^†^.

Group^‡^	Score/Level	Baseline	Month 12 (p value)	Month 24 (p value)	Month 48 (p value)
		N = 30	N = 30	N = 30	N = 20
C SCI	Motor	27.6 ± 15.6	35.3 ± 20.0 (***)	37.0 ± 19.9 (***)	36.4 ± 17.6 (***)
	Sensory				
	Light touch	55.8 ± 24.9	58.4 ± 26.5 (0.066)	59.8 ± 26.5 (*)	59.9 ± 28.8 (0.061)
	Pin prick	56.3 ± 23.4	61.9 ± 24.5 (**)	62.3 ± 24.9 (**)	62.1 ± 22.8 (*)
	Neurological	5.2 ± 1.6	6.5 ± 3.9 (**)	6.3 ± 3.7 (**)	5.4 ± 3.9 (0.180)
TL SCI		**N = 30**	**N = 30**	**N = 30**	**N = 25**
	Motor	56.8 ± 9.2	59.8 ± 9.6 (***)	60.7 ± 10.1 (***)	59.5 ± 8.5 (***)
	Sensory				
	Light touch	75.7 ± 15.7	78.2 ± 15.8 (**)	79.2 ± 15.8 (**)	78.7 ± 18.5 (*)
	Pin prick	78.2 ± 14.7	81.8 ± 16.0 (**)	82.7 ± 16.6 (***)	81.6 ± 19.9 (**)
	Neurological	18.0 ± 4.2	18.6 ± 4.2 (**)	18.7 ± 4.0 (**)	18.0 ± 4.0 (0.083)

^†^Compared with baseline, based on Wilcoxon signed rank test. Values are presented as means ± SDs.

^‡^C SCI = cervical spinal cord injury; TL SCI = thoracolumbar spinal cord injury.

*Significant at p < 0.05.

**Significant at p < 0.005.

***Significant at p < 0.0001.

**Figure 3:**
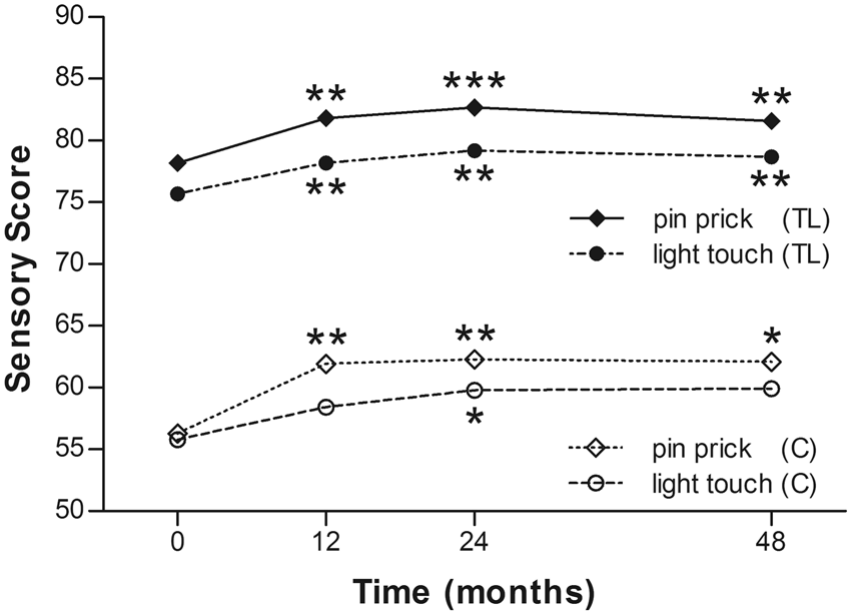
Mean ASIA sensory score (pin prick and light touch) of the 2 groups at each time point. C = cervical group; TL = thoracolumbar group; *p < 0.05; **p < 0.005; ***p < 0.0001.

The sensory scores were measured by light touch and pin prick, separately. The scores of light touch sensation in the cervical group tended to increase (from 55.8 ± 24.9 to 59.9 ± 28.8, p = 0.061), while it significantly improved from 75.7 ± 15.7 to 78.7 ± 18.5 in the thoracolumbar group (p = 0.021). The pin prick sensation improved significantly from 56.3 ± 23.4 to 62.1 ± 22.8 in the cervical group (p = 0.024), and from 78.2 ± 14.7 to 81.6 ± 19.9 in the thoracolumbar group (p = 0.002) (Table 3) (Fig. 3). Likewise, the improvement was similar to that at 24 months post operation. There was little further improvement from the post-operation 24 to 48 months.

**Figure 4:**
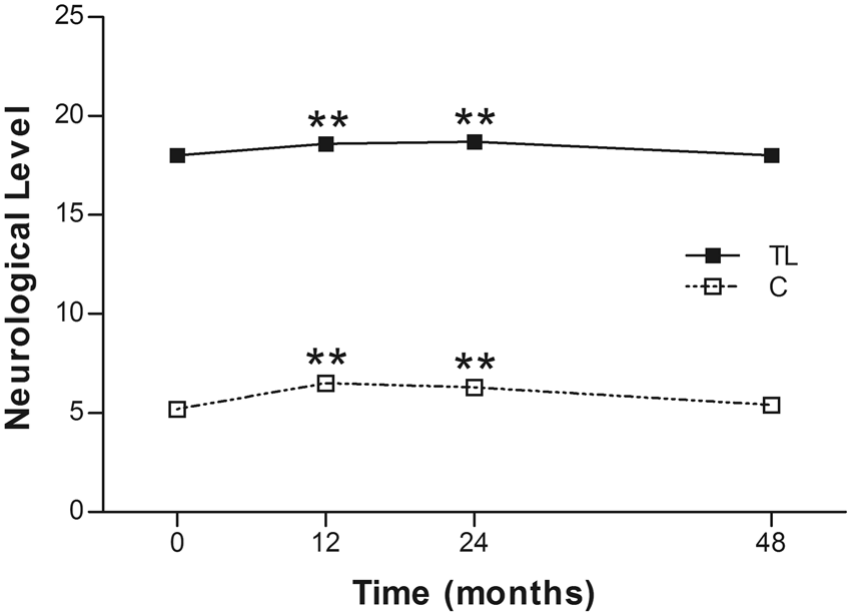
Mean ASIA neurological level of the 2 groups at each time point. C = cervical group; TL = thoracolumbar group; **p < 0.005.

The overall neurological levels improved significantly from 5.2 ± 1.6 to 6.5 ± 3.9 (*p* = 0.003) at 12 months postoperatively, and to 6.3 ± 3.7 (*p* = 0.002) at 24 months postoperatively in the cervical group. However, there was no difference at 48 months (5.4 ± 3.9, *p* = 0.180). A similar tendency was noted in the thoracolumbar group. They improved significantly from 18.0 ± 4.2 to 18.6 ± 4.2 (*p* = 0.001) at 12 months postoperatively, and to 18.7 ± 4.0 (*p* = 0.001) at 24 months postoperatively, while regressing to 18.0 ± 4.0 (*p* = 0.083) at 48 months postoperatively (Fig. 4).

### Functional Independence Measurement (FIM)

**Figure 5:**
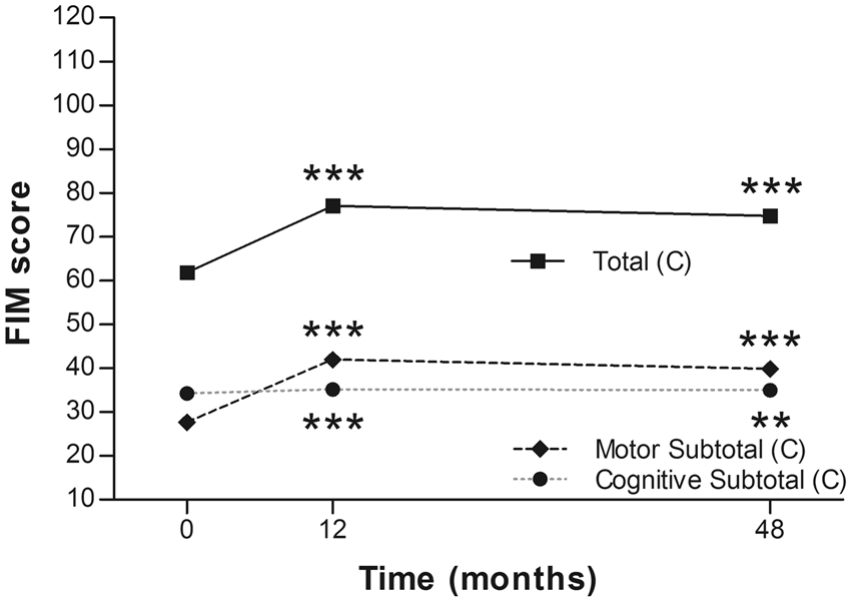
Mean Functional Independence Measure of the cervical group at each time point. **p < 0.005; ***p < 0.0001.

**Figure 6:**
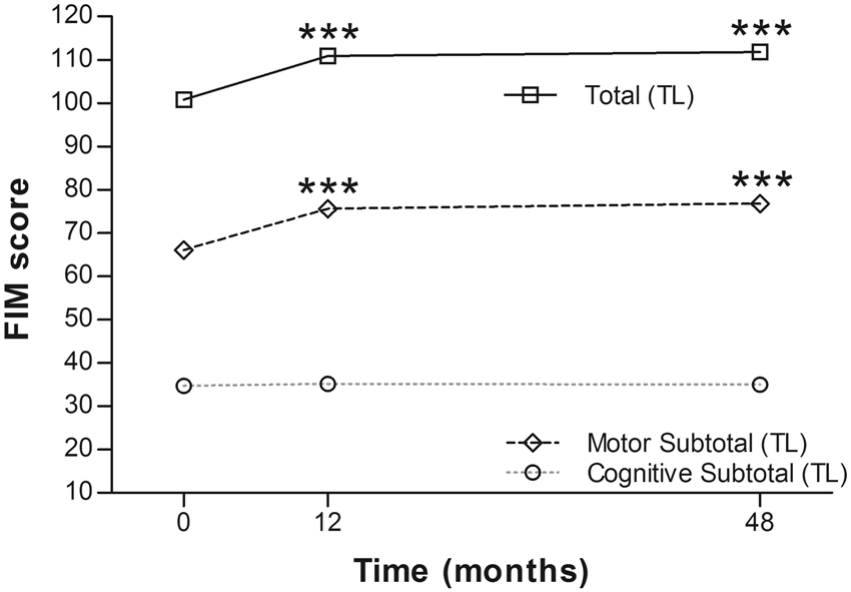
Mean Functional Independence Measure of the thoracolumbar group at each time point. ***p < 0.0001.

In both groups, the total scores and motor subtotal scores of FIM improved significantly at 48-months follow-up. Specific to the motor subtotal scores only (modified FIM for spinal cord injury), they improved from 27.6 ± 17.5 to 39.8 ± 24.1 in the cervical group (*p* < 0.0001), and from 66.1 ± 11.5 to 76.8 ± 11.2 in the thoracolumbar group (*p* < 0.0001) (Table 4) (Figs 5 and 6). The improvements were similar to that at 12 months post operation. There was no further improvement from 12 to 48 months post operation.

**Table 4:** Functional independence measure in each group^†^.

Group^‡^	Score	Baseline	Month 12 (p value)	Month 48 (p value)
		N = 30	N = 30	N = 21
C SCI	Motor subtotal	27.6 ± 17.5	41.9 ± 24.2 (***)	39.8 ± 24.1 (***)
	Cognitive subtotal	34.2 ± 1.8	35.2 ± 2.1 (***)	35.0 ± 0.0 (**)
	Total	61.8 ± 18.0	77.1 ± 25.4 (***)	74.8 ± 24.1 (***)
TL SCI		**N** = **28**	**N** = **28**	**N** = **24**
	Motor subtotal	66.1 ± 11.5	75.6 ± 11.9 (***)	76.8 ± 11.2 (***)
	Cognitive subtotal	34.7 ± 0.7	35.2 ± 1.9 (0.063)	35.0 ± 0.0 (0.063)
	Total	100.8 ± 11.7	110.9 ± 13.4 (***)	111.8 ± 11.2 (***)

^†^Compared with baseline, based on Wilcoxon signed rank test. Values are presented as means ± SDs.

^‡^C SCI = cervical spinal cord injury; TL SCI = thoracolumbar spinal cord injury.

*Significant at p < 0.05.

**Significant at p < 0.005.

***Significant at p < 0.0001.

**Figure 7:**
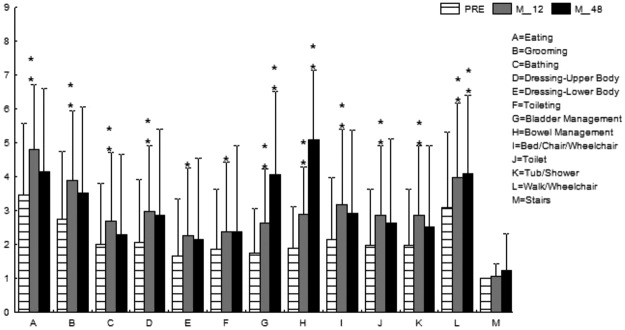
Mean Functional Independence Measure in the cervical group. *p < 0.05; **p < 0.001.

**Figure 8:**
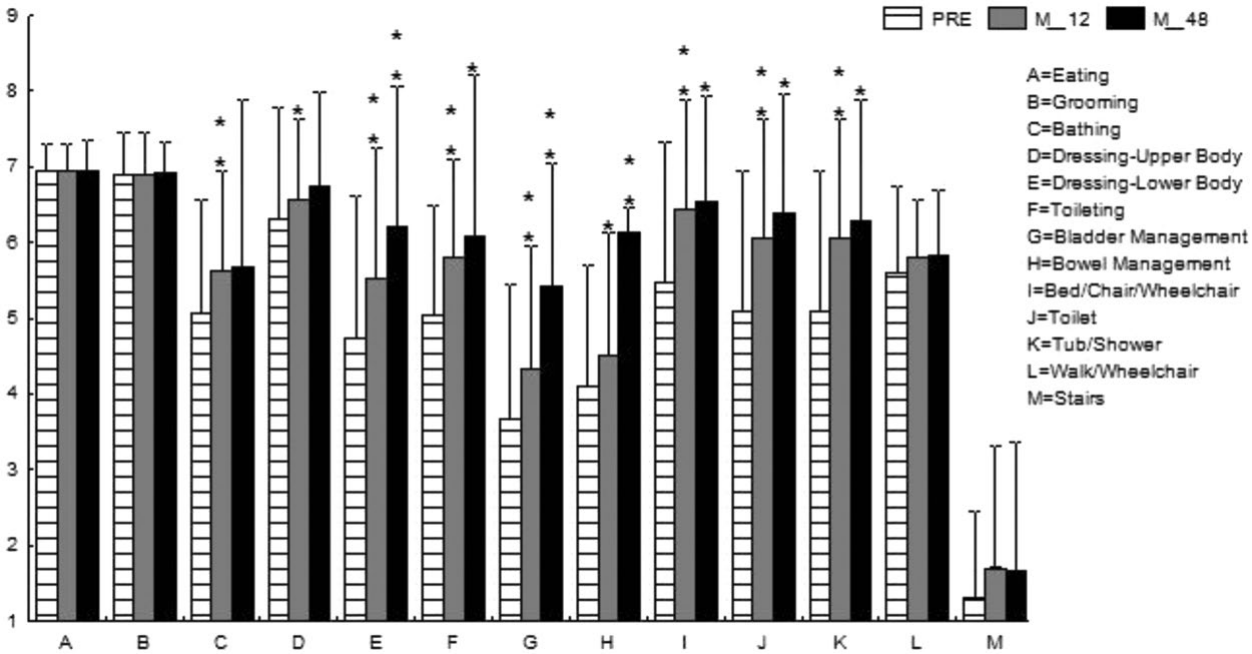
Mean Functional Independence Measure in the thoracolumbar group. *p < 0.05; **p < 0.001.

Detailed items of the modified FIM (the part of the motor subscales) are described in Figs 7 and 8 for the cervical and thoracolumbar groups, respectively. The significant improvements in the cervical group focused on sphincter control and locomotion (Fig. 7), while in the thoracolumbar group, the patients benefitted in the aspects of self-care, sphincter control, and mobility (Fig. 8).

**Figure 9:**
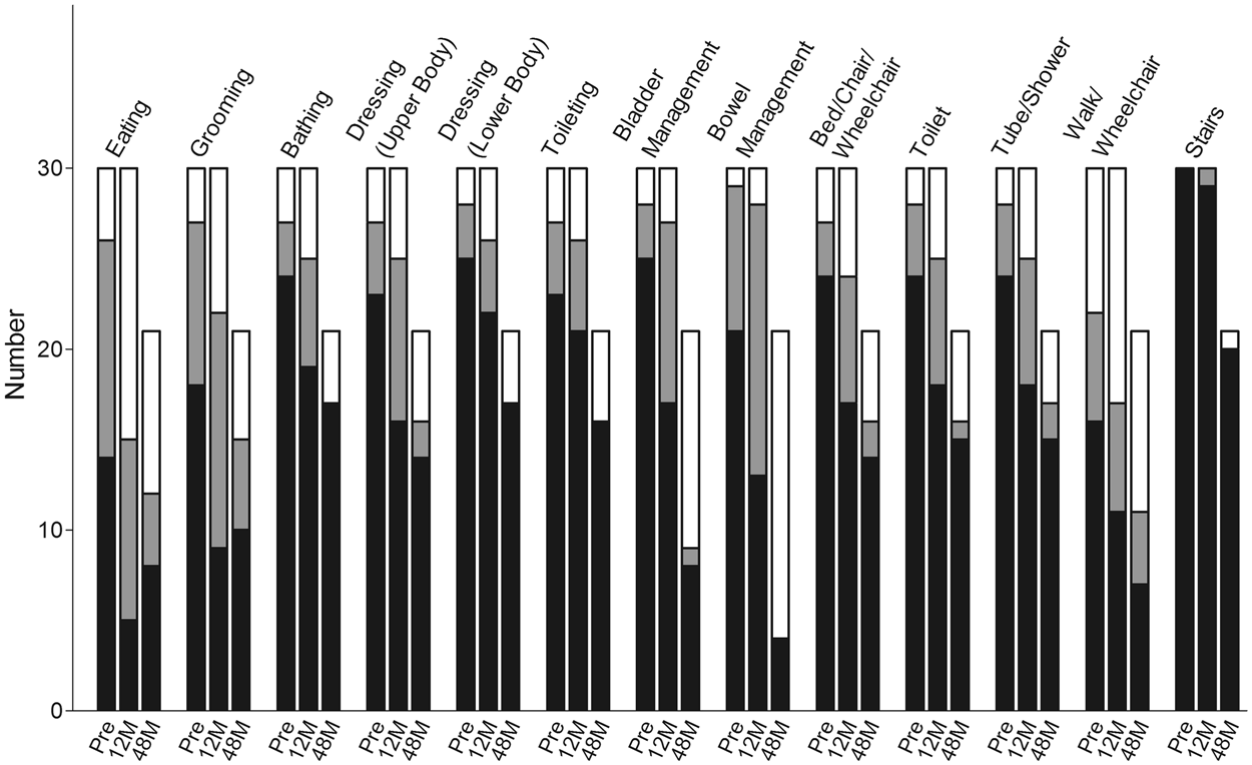
Numbers of different levels of dependence at each time point in the cervical group, translated from the Functional Independence Measure. Black bar = complete dependence on the helper; grey bar = modified dependence on the helper; white bar = no dependence on the helper.

**Figure 10:**
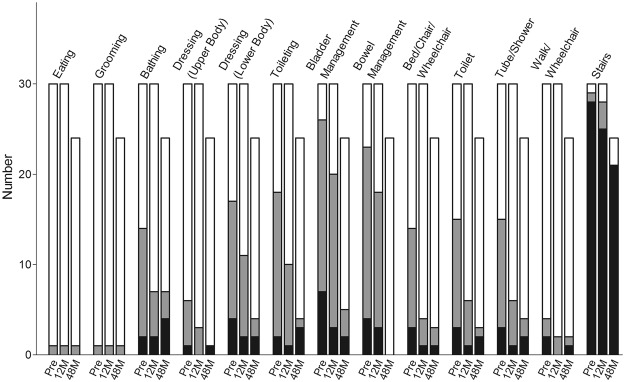
Numbers of different levels of dependence at each time point in the thoracolumbar group, translated from the Functional Independence Measure. Black bar = complete dependence on the helper, grey bar = modified dependence on the helper, white bar = no dependence on the helper.

Similar results can be brought out in another approach according to the levels of dependence on a helper. One might translate the FIM scales 1 and 2 as complete dependence on a helper, scales 3, 4 and 5 as modified dependence on the helper, while scales 6 and 7 equals no dependence on the helper^26^. By means of comparing the growth or decline of numbers in the levels of dependence at each item, one can see the progression of the patients’ daily activities as time goes by (Figs 9 and 10).

### Major adverse events

Most major adverse events occurred in the first year. There were 9 severe adverse events, including 2 mortality cases. One committed suicide 7 months after the treatment and the other died of pneumonia and sepsis 13 months after the treatment. All these severe adverse events were either “not” or “unlikely” related to the administration of aFGF. There was no new case of a major adverse event found between 13- and 48-months follow-up. Nor was there any malignancy diagnosed.

## Discussion

The clinical trial was conducted in order to test the effect of aFGF on the neurological improvement of human patients with subacute or chronic SCI, whose neurological function was at the least possibility to improve conventionally. The trial, limited by ethical issues, contained only one collection of participants and no control group with which to compare. As an extension of the previous report^23^, this paper focused on the long-term results and the late-onset of major adverse events. At 48 months after the treatment, significant improvements in the ASIA Impairment Scale, motor score, sensory score, and modified FIM were noted in the 46 subjects. There was no major adverse event related to the treatment, nor malignancy.

Spinal cord injury causes considerable impact on health, which hardly diminishes over time^28^. It was not until 1996 that Cheng *et al*. suggested autologous peripheral nerve grafting stabilized with fibrin glue containing aFGF as a possible repair strategy for completely transected spinal cords in rats^4^. It was for the first time that central nervous axonal regeneration became possible. The repair strategy was reported as successful for complete spinal cord transection in other mammal animal studies^9,12^. This repair strategy benefits humans, too. A young man with chronic paraplegia due to spinal cord hemisection from a stab injury 3 years previously was reported. He obtained significant improvement in the ASIA Impairment Scale, the motor score, sensory score, and functional status (from being wheelchair bound to ambulatory independence with a walker) 2.5 years after the therapy strategy^16^. Nevertheless, there is a rare chance to meet the spinal cord discontinuity in the clinical scenario. Therefore, the majority of the patients in this trial received the treatment of direct application of aFGF in fibrin glue without peripheral nerve grafting.

In the current study, four of 21 (19%) patients in the cervical SCI group and 13 of 25 (52%) in the thoracolumbar group obtained at least 1-grade improvement on the ASIA Impairment Scale, including 2 (out of 8 cases of Grade A in the cervical group) gaining a 2-grade improvement (from Grade A to Grade C) (Table 2). In another large-scale study of traumatic SCI, it was concluded that only 5.6% of cases of complete injury at 1 year post injury converted to incomplete, while only 2.1% of cases converted to motor incomplete status at 5 years^29^. Compared to that report, the improvement rate of the current study is apparently superior. As well as the ASIA Impairment Scale, the authors also noted improvements in motor scores and sensory scores in both groups (Figs 2 and 3). The overall neurological level improved significantly at the time points of 12 and 24 months, but not at 48 months (Fig. 4). The neurological level was calculated according to crucial criteria filling a normal functional (both motor and sensory) segment; therefore the range of change was so small. The regression at 48 months is probably due to the loss of patient follow-up since the decrease of cases might interfere with the statistical data. However, the objectives did present progression in daily activities, revealed by the increase in the mean motor subtotal scores of the FIM (Figs 5 and 6) and of mean scores of specific items of the FIM (Figs 7 and 8), and the decline in patient numbers with dependence on helpers (Figs 9 and 10). Nevertheless, when interpreting the positive results, one should keep in mind that the achievements might come from the treatment strategy, rehabilitation program, or the synergy of both.

The neurological improvement was significant at 24 months post operation, when compared to that at pre-operation. In this report, clearly the effect had been maintained from the end of the 2^nd^ year to the completion of the 4^th^ year, without additional boosters of aFGF. Although there was no further improvement of the neurological function from 24 to 48 months, the effectiveness of continuous exertion of the rehabilitation programs should not be neglected.

Except for the inherent morbidities of spinal cord injury, such as urinary tract infection, pressure sores, and pneumonia, the adverse events associated with the repair therapy draw more misgivings. In this study, there was no complication directly caused by the operation. Concerning the administration of the therapeutic cocktail, no malignancy was found throughout the 4-year follow-up. These results probably eliminate the suspicions about the carcinogenicity of aFGF, and further enhance the feasibility of this repair strategy.

The positive results presented here should be interpreted with caution for several reasons. First, the present study lacked a control group due to ethical issues and strict inclusion/exclusion criteria. Second, the neurological improvement may come from not only the effect of aFGF, but also the surgical procedures of neurolysis, or the rehabilitation program. Third, one’s intrinsic capacity to repair the injured nervous system cannot be neglected. The study presented here is to offer valuable data for further scientific research. Obviously, there are more questions raised than answered.

## Conclusions

This open-label clinical study demonstrated the safety and feasibility of using aFGF for human SCI. There were significant improvements in the ASIA Impairment Scale, the motor score and sensory score at 48 months after the repair strategy. Despite no full recovery, the quality of daily life of the SCI patients improved, as the modified FIM suggested. There were no clinically related major adverse events throughout the 4 years follow-up. Further large-scale, randomized, controlled investigations are needed to evaluate efficacy and feasibility.
